# Supplementary material for: The Coverage and Acceptance Spectrum of COVID-19 Vaccines among Healthcare Professionals in Western Tanzania: What Can We Learn from This Pandemic?
Source: Vaccines (Basel). 2022 Aug 30;10(9):1429. doi: 10.3390/vaccines10091429 (PMC9503367; doi:10.3390/vaccines10091429)
Supplement: Supplementary file 1 [file vaccines-10-01429-s001.zip › vaccines-1841447-supplementary.pdf]

Table S1: Distribution of 811 health professionals from health facilities involved in the study

District name	Health facility	Number of *HP	Percentage
Mwanza city	Bugando Medical Centre (Tertiary Hosp)	233	28.73
	Sekou-toure Regional Hospital	132	16.28
Ukerewe	Nansio district hospital	73	9.00
	Kagunguli health center	35	4.32
	Muriti health center	32	3.95
	Nakatunguru health center	22	2.71
	Murutunguru dispensary	12	1.50
	Bugorola dispensary	11	1.40
	Bulamba dispensary	3	0.4
	Hamyebe dispensary	2	0.3
	Muruseni dispensary	2	0.3
	Buzengwe dispensary	1	0.1
	Hamkoko dispensary	1	0.1
Magu	Magu district hospital	80	9.86
	Nyanguge health center	25	3.1
	Kahangara health center	24	2.9
	Kisesa health center	19	2.3
	Lugeye health center	12	1.5
Misungwi	Misungwi district hospital	41	5.1
	Koromije health center	17	2.1
	Bukumbi district hospital	16	1.9
	Ibongoya dispensary	11	1.4
	Idetemya dispensary	7	0.9
**TOTAL**	**ALL SITES**	**811**	**100**

*HP: Health professionals
